# Reply to letters on review ‘Evolving treatment strategies for myeloma’

**DOI:** 10.1038/sj.bjc.6602695

**Published:** 2005-07-12

**Authors:** G J Morgan, F E Davies

**Affiliations:** 1 Royal Marsden Hospital and Institute of Cancer Research, Downs Road, Surrey SM2 5PT, UK

**Sir**,

We would like to thank the authors for their comments on our recent review article ‘evolving treatment strategies for myeloma’. Although very rare, the occasional patient may present with a pyrexia of unknown origin (PUO), arising presumably from cytokines released by the myeloma plasma cells. While we agree this is possible, it should not be the first conclusion as the immunosuppressed myeloma patient is at great risk of infection. These patients represent a difficult diagnostic challenge and need to be screened for active infection and treated with antibiotics where appropriate. The only clear proof that the temperature is related to the myeloma is that it will resolve when antimyeloma treatment is initiated. However, it is important not to delay the initiation of treatment, which may be detrimental to the patient. Therefore, we totally agree that once active infection had been ruled out in a newly diagnosed patient with a PUO, chemotherapy should be initiated promptly.

The antitumour role of bisphosphonates in myeloma is intriguing especially with the highly potent third-generation compounds such as zoledronic acid, which are now in routine use. To date, their antimyeloma effect has only been shown in cell lines and mouse models. The apoptotic action of sodium clodronate is clearly different from that of zoledronic acid. Clodronate is metabolised to a number of ATP analogues, which are resistant to hydrolysis and therefore accumulate within the cell resulting in the inhibition of important metabolic enzymes, such as phosphatases and pyrophosphatases, whereas zoledronic acid inhibits the mevalonate pathway leading to an inhibition of the prenylation of small GTPases important in cell signalling, such as RAS. While these and other effects such as inhibition of *γδ* T cells are clearly demonstratable *in vitro*, their relevance *in vivo* is less clear. Dose levels in this setting are lower, and the drugs rapidly leave the circulation and become fixed in the bone; thus, it has to be postulated that bisphosphonates are concentrated in proximity to osteoclasts and can reach doses that are able to kill them. The relevance of this mechanism to myeloma plasma cells rather than osteoclasts is less certain. However, what is clear is that the direct inhibition of bone resorption and the resulting change in the pattern of cytokines produced can inhibit positive feedback loops important in maintaining survival of the myeloma clone.

Available data from numerous clinical trials only support the effect of bisphosphonates in inhibiting bone resorption and reducing skeletal-related events, and we believe that no conclusion regarding their *in vivo* antimyeloma effect can be drawn at present. We agree in view of the dramatic effects on skeletal-related events that all patients should receive a bisphosphonate; however, whether this should be a first- or third-generation derivative is unclear. We eagerly await the results of the current large Medical Research Council randomised phase III trial, which compares oral sodium clodronate with intravenous zoledronic acid in order to determine whether there is any differential antimyeloma effect in patients.

